# Increased heat tolerance of geothermal plants at the cost of reduced performance under cooler conditions

**DOI:** 10.1186/s12862-025-02422-7

**Published:** 2025-08-14

**Authors:** Jan-Niklas Nuppenau, Johan Ehrlén, Aelys M. Humphreys

**Affiliations:** 1 Department of Ecology, Environment and Plant Sciences, Stockholm University, Stockholm, Sweden; 2 Bolin Centre for Climate Research, Stockholm University, Stockholm, Sweden

**Keywords:** *Agrostis*, Biomass, Cold tolerance, Generalist-specialist trade-off, Growth, Hot-cold trade-off, Survival, Temperature stress, Thermal tolerance, Vitality

## Abstract

**Background:**

All plants are influenced by the temperatures they are exposed to and fascinating adaptations to extreme temperatures have been described for many of them. However, the extent to which adaptation to thermal extremes is associated with costs, in terms of reduced performance at less or other stressful temperatures, is poorly known, especially for plants. In Iceland, there are two lineages of *Agrostis stolonifera*, one that occurs exclusively on geothermally heated soils (> 50 °C) and one that is only found on non-thermal soils. Since Iceland is a subarctic island, non-thermal areas surrounding the geothermal areas can get bitterly cold. This stark contrast in temperatures over short geographic distances provides an excellent system for studying adaptations to thermal extremes and potentially associated trade-offs. To test whether the geothermal lineage is more heat tolerant and whether this heat tolerance is associated with reduced performance under cooler conditions, we compared the heat and cold stress responses of the two lineages experimentally.

**Results:**

No plants survived the hottest treatment (56 °C), only geothermal plants survived the second hottest treatment (49 °C) and geothermal plants also outperformed the non-thermal plants following the 46 °C treatment. In contrast, there were no differences in survival between geothermal and non-thermal plants under intermediate and cold conditions (41 °C, 21 °C and − 4 °C), but non-thermal plants outperformed geothermal plants under these conditions.

**Conclusions:**

These results suggest that there is a trade-off between tolerating extreme heat and performance under cooler conditions, possibly indicating that geothermal *A. stolonifera* represents a specialised thermophilic lineage in Iceland. Our findings provide new empirical data on whole-plant responses to different thermal conditions, further understanding of the consequences of adapting to high and low temperature extremes, and raise new questions about the mechanisms, benefits and costs of thermal specialisation under different climatic conditions.

**Supplementary Information:**

The online version contains supplementary material available at 10.1186/s12862-025-02422-7.

## Background

Temperature affects life at all levels, from the speed of chemical reactions to the timing of life history events and the distribution of biodiversity within and among biomes [1–4]. However, while several studies have shown that organisms are generally adapted to the temperatures they experience in their natural habitat [3, 5–7], a major discussion revolves around the level of thermal specialization they evolve [2, 8]. The general idea is that thermal specialists are expected in stable thermal environments, whereas generalists are favoured in unstable environments [8–11]. However, empirical support for the level of thermal specialisation in relation to the environmental stability is mixed. For example, broad geographic patterns show the expected wider thermal tolerance breadth (range of temperatures over which an organism can function) in more variable environments, such as at high latitudes [e.g. 12, 13], while local adaptation of populations to differing temperatures has been shown in many other cases [e.g. 14–18]. However, local adaptation rarely leads to specialisation and thermal specialists are relatively rare, indicating that a number of factors can influence or hinder thermal specialisation [3, 19, 20]. Overall, our understanding of patterns of thermal adaptation in relation to the local environment is limited, especially for plants [3, 21, 22].

In a laboratory setting, there has been much progress over the last decades in understanding plant adaptations to upper [e.g. 23–25] and lower [e.g. 26–28] thermal extremes. However, most of this research has focussed on adaptation to either high or low temperature, with a recent estimate suggesting only 5% of studies address both high and low temperature responses [29]. As a consequence, much less is known about whether or how adapting to one thermal extreme affects adaptation to the other thermal extreme or to less extreme thermal conditions (i.e. performance under more optimal conditions). The stresses caused by high and low temperatures elicit different physiological and metabolic responses, e.g. production of heat shock proteins in response to heat versus synthesis of ice-nucleating substances and ice-binding proteins in response to freezing [24–27, 30]. They are therefore expected to come at a cost in terms of resources and can trade off against each other, e.g. via genetic correlations/constraints, or biochemical trade-offs [22, 31, 32]. Accordingly, a number of hypotheses have been developed regarding how such trade-offs could influence variation in thermal adaptation. One is the ‘hot-cold trade-off hypothesis’ that posits that there should be performance trade-offs between hot and cold environments [22, 33]. This means cold specialists are expected to evolve in cold environments and warm specialists in warm environments, and that being adapted to one thermal extreme limits or prevents adaptation to the other. A second hypothesis is the ‘specialist-generalist trade-off hypothesis’ that posits that an increase in the range of temperatures over which an organisms can function comes at the cost of decreased maximum performance [22, 32]. This means that organisms adapted to both hot and cold environments perform poorly in both, suggesting a ‘jack of all trades, master of none’ scenario [22, 34]. There is limited support available for either of these hypotheses in plants [22, 34].

One approach for testing these hypotheses is thermal performance curves (TPCs), models displaying unimodal, continuous reaction norms of performance across a temperature range [3, 22, 35, 36]. These are widely used in zoology [33, 37, 38], and to some degree also in plants, mainly based on photosynthetic performance [e.g. 35, 39]. However, only a few empirical TPCs from fitness-based measurements, or traits that approximate fitness such as germination, survival or growth rates, have been quantified for plants [22]. For example, a broader TPC (i.e. wider thermal performance breadth) has been shown to correlate negatively with maximum performance (growth) at the optimal temperature for *Mollugo verticillata* [39]. Still, the level of support for the hypotheses outlined above in nature, i.e. whether adapting to thermal extremes is generally associated with reduced maximum performance (specialist-generalist tradeoff) or whether adapting to one thermal extreme generally limits adaptation to the other (hot-cold tradeoff), is largely unknown for plants.

Geothermally heated areas offer excellent opportunities for studying adaptation to thermal extremes and associated trade-offs. In geothermal areas, some plants grow at temperatures that would be scorching to most life [40–42]. However, the heat generated belowground soon dissipates aboveground so air temperatures and adjacent non-thermal areas can get very cold, especially for geothermal areas found at high latitudes or elevations [43, 44]. The resulting temperature gradient, from scorching hot (up to 90 °C) to bitterly cold (down to −10 °C) over short geographic distances [44, 45] mitigates many of the issues inherent in studying climatic gradients over larger geographic distances (e.g. confounding effects of factors other than temperature), making geothermal systems ideal for studying potential trade-offs involved in adapting to thermal extremes.

Most research on geothermal plants to date has focused on adaptations to extreme heat and responses to elevated temperatures [e.g. 40, 46–51]. For example, it has been shown that some geothermal plants have altered thermoregulation and increased thermal tolerance [47], some have undergone phenological shifts and flower earlier [49], others have undergone life history shifts and behave as annuals [50] and others still have special symbioses with microbes that help them tolerate the hottest temperatures [40, 51]. However, the effect of adaptation to geothermal heat on performance under other (i.e. cold or freezing) or less (i.e. optimal) extreme conditions has received little attention, besides a study on snails [37].

Here, we studied the grass species *Agrostis stolonifera* (Poaceae) from geothermal and non-thermal areas of the subarctic island Iceland to test for trade-offs in adapting to thermal extremes, such that being adapted to geothermal heat is associated with reduced performance under cold (< 10 °C) and intermediate (10–30 °C, the temperature range for optimal growth for most plants [52]) conditions. *Agrostis* is especially interesting for studying adaptation to geothermal areas as it is known to form ecotypes in response to special edaphic conditions, such as mining sites and salinity [53, 54]. The genus as a whole is distributed throughout the Northern Temperate zone [55–57], and it is assumed therefore that most of its constituent species were ancestrally adapted to long, cold winters. *Agrostis stolonifera* itself is known to be very winter hardy [58]. A recent phylogeographic study found that geothermal and non-thermal populations of *A. stolonifera* form two separate lineages in Iceland: one that exclusively occurs in the hottest geothermal areas (the geothermal lineage; found at soil temperatures reaching 70 °C) and one that occurs in non-thermal areas in Iceland, Norway and Greenland (the non-thermal lineage) [45]. The two lineages are not each other’s closest relatives, suggesting that *A. stolonifera* colonized Iceland at least twice and that the two lineages have experienced starkly different temperature conditions over multiple generations.

We hypothesized that the geothermal lineage of *A. stolonifera* is more heat tolerant than the non-thermal lineage, and that this increased heat tolerance is associated with reduced performance under cold and less extreme conditions. If true, this would suggest that geothermal *A. stolonifera* is a thermophilic specialist, supporting the hot-cold trade-off hypothesis. Alternatively, if geothermal *A. stolonifera* performs worse than non-thermal *A. stolonifera* across a broader range of temperatures, this would suggest it is a generalist, supporting the specialist-generalist trade-off hypothesis. To test our hypotheses, we experimentally compared the performance of the two Icelandic lineages to a range of temperatures; four heat treatments, one cold treatment and one at intermediate conditions within the optimal temperature range for most plants. Thus, we did not use a full TPC approach but instead measured sensitivity to each temperature treatment using the fitness components survival and growth, assessed as aboveground biomass production and vitality, i.e., the number of new leaves produced post treatment. More specifically, we predicted that, compared to the non-thermal lineage, the geothermal lineage has (i) higher survival rates and performance (as measured by biomass and vitality) following heat treatment. We further predicted that this comes at a cost that reduces performance under cooler conditions, such that the geothermal lineage has lower survival rates and performance (ii) following cold treatment and (iii) at intermediate temperatures.

## Methods

### Study system

**Fig. 1:**
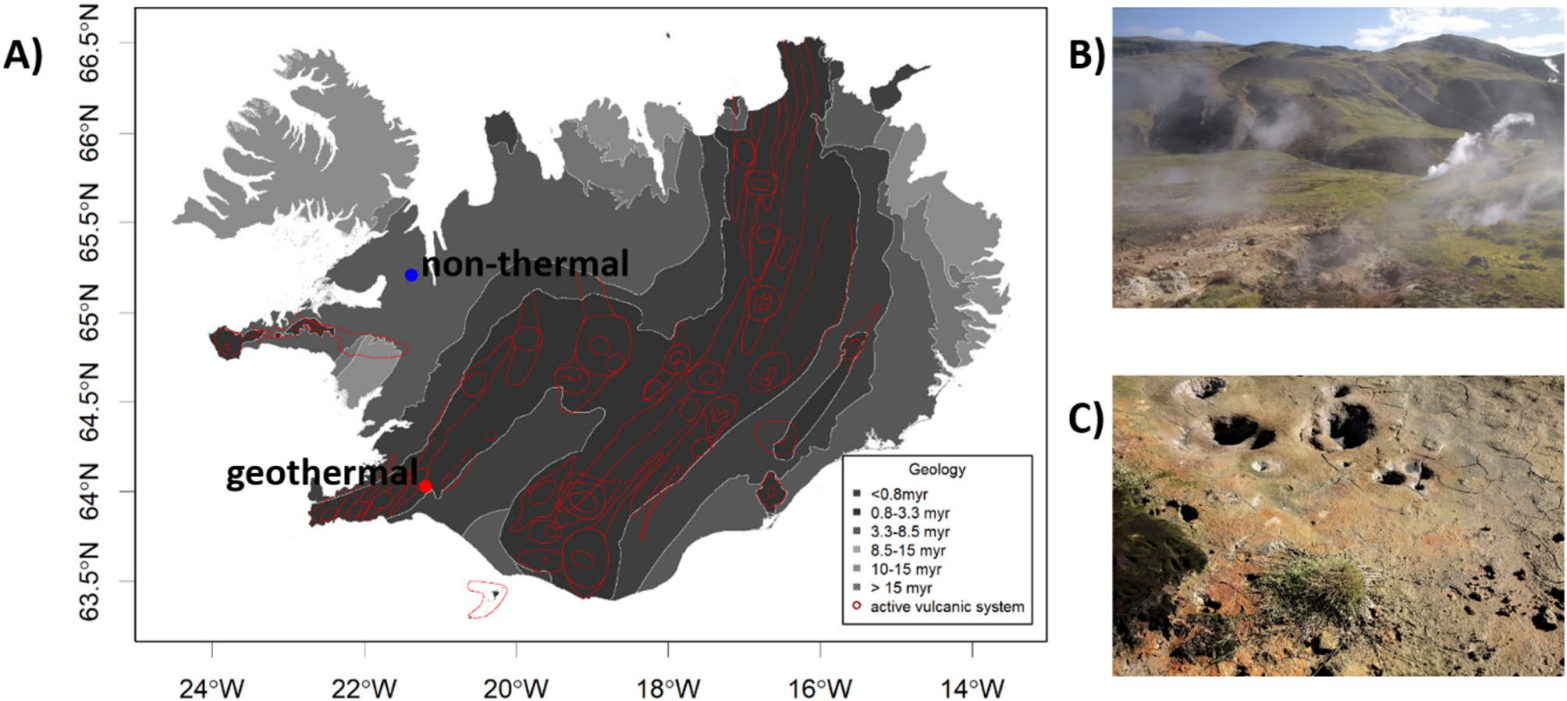
*Agrostis stolonifera* in Iceland. **(a)** Distribution of geothermal (red) and non-thermal (blue) study sites in Iceland, shown with the age of the underlying bedrock (greyscale), as well as current active volcanic systems (red lines). Tectonic layers were taken from [97]. **(b)** The geothermal area at Hengill. **(c)** *Agrostis stolonifera* growing in the geothermal area close to a fumarole. For a picture of the non-thermal area see Fig. S1 in the supplement

To test for differences in sensitivity to heat and cold treatment, we grew plants from seeds collected in Iceland from a geothermally heated area (representing the geothermal lineage) and a non-heated area (representing the non-thermal lineage) [45]. The geothermal area is located 150 m above sea-level in the Grændalur valley in the Hengill volcanically active area (64.01’48"N, 21.11’57"W, Fig. 1a). The Hengill central volcano hosts one of the most powerful geothermal fields in Iceland [59]. It is not clear how stable geothermal areas in Iceland are and since when they have existed but geothermal activity has been reported at Hengill for at least 50 years (probably for much longer) [60]. The geothermal area lies on a southeast-facing hillside covered by dense meadows dominated by grasses, with numerous fumaroles and small, heated streams (Fig. 1b). Close to the fumaroles the soil is often bare, with only scattered *A. stolonifera*,* Thymus praecox* and mosses (Fig. 1c). The non-thermal area is located 105 m above sea level, about 30 km inland (65.12’24"N, 21.22’55"W; Fig. 1a), on a southeast-facing hillside with wet, sandy soil, next to a small creek (Fig. S1). The vegetation is rich in mosses, Cyperaceae and Poaceae.

To describe the thermal conditions at the geothermal and non-thermal study sites, and how they change over the course of a year, we logged temperatures between September 2018 and September 2019. At each site, one logger (EL-USB2, Lascarelectronics) was placed just above ground level and another (iButton DS1922, Maximintegrated) was placed belowground at 10 cm depth, close to a healthy looking *A. stolonifera* plant and with temperatures logged every two hours. Unfortunately, the belowground logger at the geothermal site was pulled out of the ground on the 5th of November 2018 (after which it remained on the soil surface logging aboveground temperatures) and the original aboveground logger stopped recording at the end of October 2018 (Fig. S2). We summarised the data that were successfully logged (1 month belowground, 1 year aboveground) using the ‘biovars’ function in the R [61] package *dismo* [62]. These data showed that the geothermal area was warmer on average (40.7 vs. 4.7°C belowground and 11.1 vs. 4.5°C aboveground), being hotter in summer and milder in winter, but also much more variable (Table 1, Fig. S2). The geothermal area regularly experienced >50 °C (maximum recorded temperature was 56.5°C belowground and 53.5°C at the soil surface), whereas the maximum temperatures recorded at the non-thermal site were 12.6°C belowground and 33 °C aboveground (Table 1, Fig. S2; note that the maximum aboveground temperatures might be slight overestimates of the temperatures experienced by the plants as no evapotranspiration occurs close to the sensor). The duration of these maximum temperatures lasted from a few hours to a couple of days. Some of the temperature fluctuations at the geothermal area were independent of diurnal variation (Fig. S2). Finally, freezing temperatures occurred several times during winter at both the geothermal and non-thermal sites, sometimes for several days. The minimum aboveground temperature recorded at the geothermal site was − 8.5°C compared to −12°C at the non-thermal site. During summer, sub-zero temperatures were recorded aboveground in the non-thermal area but not the geothermal area.

**Table 1:** Temperature conditions in the geothermal and the non-thermal areas based on seven bioclimatic variables summarised from temperature data logged in the field (September 2018–September 2019) using the bioclim variables of Hijmans et al. [96]

Area	Logger placement	BIO1	BIO4	BIO5	BIO6	BIO7	BIO10	BIO11
Non-thermal	Belowground	4.71	320.16	12.58	0.54	12.04	8.99	1.13
Non-thermal	Aboveground	4.52	653.07	33.00	−12.00	45.00	13.50	−1.50
Geothermal^1^	Below ground	40.70	838.90	56.51	12.56	43.95	NA	NA
Geothermal^2^	Aboveground	11.13	1071.65	53.52	−8.50	62.02	20.82^3^	3.31^4^

^1^Based on data logged during 15 September 2018–5 November 2018

^2^Based on data logged during 5 November 2018–17 September 2019

^3^Based on data logged during four summer months (May 2019– August 2019)

^4^Based on data logged during four winter months (November 2018– February 2019)

BIO1 = Mean temperature for the period of monitoring, BIO4 = Temperature seasonality (standard deviation × 100), BIO5 = Maximum temperature of warmest month, BIO6 = Minimum temperature of the coldest month, BIO7 = Temperature range for the period of monitoring (BIO5-BIO6), BIO10 = Mean temperature of the warmest quarter, BIO11 = Mean temperature of the coldest quarter. The unit for the bioclimatic variables is °C

### Plant material

Most of the plant thermal tolerance literature is based on the temperatures at which leaves are damaged and photosynthesis disrupted [13, 29, 47, 63, 64]. Fewer studies quantify whole-plant responses under different thermal conditions [22]. For certain organisms (e.g. long-lived, large trees) this may not be feasible, but for other organisms (e.g. herbaceous plants such as grasses) or developmental stages (e.g. seedlings) there are clear benefits to studying whole plant responses over excised plant parts and tissues. For example, cut shoots or leaves have been found to have both higher and lower frost resistance than attached ones [65], which can complicate extrapolating plant-part measures to plant responses in nature. Since grasses are amenable to measuring whole plant responses, we took this approach here.

Seeds were collected in the field in September 2018. At the time of seed collection, soil temperature was measured at 10 cm depth at all sampling localities and, at the geothermal area, seeds were only collected from plants growing in soil > 20 °C. Soil temperatures at the geothermal site were up to 69 °C at 10 cm depth on the day of seed collection [45], compared to around 5 °C at the non-thermal site. At each site, mature seeds were collected from at least 10 plants distributed over the whole area. Seeds were placed in paper bags and stored in a dry, dark place.

Sets of genetically identical propagules (stolons) harvested from one-year-old plants were used in the experiment. These plants were grown from the field-collected seeds, which were germinated at 26 °C in a mixture of fine sand and moist potting soil in spring 2019. Seedlings were transferred to bigger pots (10 × 10 × 10 cm³) after eight weeks, when they were placed outside in a common garden until autumn 2020 (i.e. including winter). Plants were regularly watered during the whole growth period (i.e. not in winter). In autumn 2020, 15 plants of geothermal origin and 15 plants of non-thermal origin were selected at random. Offshoot plants were harvested from their stolons and rooted in water in glass vases for 20 days (Fig. S3). After rooting, six off-shoot plants of a similar size (leaves 4–6 cm long, roots 2–4 cm long) were selected from each parent plant to create six replicates (clones) of each genetically different individual (parent) for the experiment. This resulted in 6 *x* 15, i.e. 90 plants in total of geothermal origin and 90 plants of non-thermal origin. The selected plants were transferred to pots (diameter: 6.6 cm, height: 7 cm) with a 50:50 mixture of coarse sand and potting soil. Plants remained in these pots in the greenhouse at constant long-day light conditions (12 h light, 12 h dark) and an intermediate temperature of 21 °C for a five-month initial growth period (Fig. S3). As the natural habitat of *A. stolonifera* is very wet soils, all plants were kept in water-saturated soil throughout the cultivation period and experiment. Before the start of the experiment, all plants were checked for signs of wilting, root boundedness or damage. All plants looked healthy, apart from four plants that died, two of geothermal and two of non-thermal origin. Finally, we checked whether size differences among plants at the end of the 5-month growth period differed between geothermal and non-thermal plants. They did not markedly differ (not shown) and should therefore not bias the results. However, we still accounted for any size differences by including two measures of performance: biomass and “vitality”, based on a visual regrowth score, which is less dependent on size than biomass (see below).

### Thermal experiment

After five months of cultivation, replicate plants were divided into six groups: four were exposed to heat treatment (24 h at 41, 46, 49 and 56 °C in controlled conditions), one to cold treatment (−4–0 °C during two consecutive frost nights) and one was kept in the greenhouse at 21 °C (intermediate growth temperature; 12 h light, 12 h dark, Table S1, Fig S4). The intermediate temperature (21 °C) lies within the range for optimal growth (10–30 ∘C) for most plants [52], and is regularly experienced by both lineages in Iceland [66]. The heat and cold treatments lie outside this range and should thus induce heat and chilling/freezing stress responses, respectively [67]. Plants were not acclimated prior to the heat and cold treatments but the temperature increase/decrease to the final treatment temperature was gradual (see below). The short exposure times were chosen to reflect the temperature fluctuations observed at geothermal areas and during cold winter nights (Fig. S2).

The temperature range for the heat treatments was based on the temperature at which protein degradation and heat shock protein synthesis are induced in *A. stolonifera* (40–45 °C) [68], and on the highest aboveground and belowground temperatures measured in the field at the geothermal site (53.5 and 56.5 °C; Table 1). Plants were placed in an oven as it was switched on (one replicate at a time). The initial temperature was thus room temperature and the oven needed between two (41 °C) and nine (56 °C) hours to reach the final temperature (Fig. S4), which allowed for some acclimation and development of an immediate heat shock response [69]. The oven had no lighting, meaning the plants were in the dark during the 24 h of the heat treatment. Relative humidity and temperature were measured with an EL-USB2 logger in the oven throughout each treatment. The humidity did not drop below 40% over longer periods of time in any treatment; thus the heat treatment did not cause drought stress or root desiccation (Fig. S5).

As even mild frost exposure can be detrimental to most plants in their non-hardened state, including grasses [13, 70, 71], the cold treatment was implemented outdoors, when there were night frosts but non-freezing daytime conditions (in April 2021). Plants were kept in their pots and placed outside in a meadow close to Stockholm University in the late afternoon. During the first night, soil temperatures reached the minimum of −3 °C for only 1.5 h, so the plants were kept outside for a second night (minimum − 4 °C and below − 3 °C for at least 6 h; Fig. S4) before being returned to the greenhouse. Plants experienced natural light conditions (15 h light, 9 h dark), and daytime soil temperatures reached 28 °C as it was sunny and the plants were not sheltered.

Each treatment included 15 genetically different individuals from the geothermal site and 15 genetically different individuals from the non-thermal site, except the 49 and 56 °C treatments that were performed on 14 individuals (Table S1). The same genetic individual was exposed to all temperature treatments (Fig. S3). Plants were exposed to darkness during all treatments but the heat treatments were the only ones performed entirely in the dark (i.e. plants were exposed to darkness for 9, 12–24 h, depending on the treatment; Table S1). All plants were accustomed to dark during the initial growth period.

Following treatment, plants were brought back to the greenhouse (21 °C; 12 h light, 12 h dark) and all plants, including those that remained at 21 °C throughout the experiment, were cut to a height of 10 cm. They were then left at 21 °C for a 30-day regrowth period, following which responses were measured as whole-plant survival and, for surviving indiciduals, dry mass and vitality. Survival at the end of the 30-day period was assessed as a binary response (alive or not) based on visual inspection. Biomass was measured as the aboveground regrowth following the 30-day regrowth period. All fresh growth was cut, dried for 48 h at 70 °C and then weighed using a Buch & Holm GF-300 scale. Vitality was scored visually on a scale from 1 to 7, using a modification of Larsen’s [72] classic method (where 1 = old growth still green but no fresh (new) leaves, 2 = minimal fresh leaves, 3 = one proper fresh leaf, 4 = 2–3 fresh leaves, 5 = 4–5 fresh leaves, 6 = more than 5 fresh leaves, 7 = old growth still green + plenty fresh leaves). Regrowth was scored by the same person for all plants.

### Statistical analyses

All statistical analyses were performed using R [61].To test for effects of plant origin (equivalent to the geothermal vs. non-thermal lineage) on sensitivity to different treatments, we first conducted pairwise tests between geothermal and non-thermal plants for each temperature treatment. To test hypothesis (i), that geothermal plants have higher survival and performance following heat treatment, we compared survival, biomass and vitality between geothermal and non-thermal plants for each heat treatment. To test hypothesis (ii), that geothermal plants have lower survival and performance following cold treatment, we compared survival, biomass and vitality between geothermal and non-thermal plants following cold treatment. To test hypothesis (iii), that geothermal plants have lower performance at intermediate conditions, we compared biomass and vitality between the geothermal and non-thermal plants kept at the intermediate growth temperature. We also tested, for plants of geothermal and non-thermal origin separately, how plants were affected by the heat and cold treatments relative to the intermediate conditions using pairwise tests of responses to the different treatments (survival, vitality and biomass) compared to the responses to intermediate conditions. This is equivalent to testing the effect of a treatment relative to a control, because in contrast the plant exposed to the heat and cold treatments, the plants kept at 21 °C were not exposed to a short-term temperature change. For survival, we used Fisher’s exact test. For biomass of surviving individuals, we used a student’s T-test after square-root transformation to achieve normality. The T-test is a specific type of GLM particularly useful for comparing the means of two groups. Therefore, we chose it as a simple test for assessing whether the means of two normally distributed groups differ from one another (the square-root transformed biomass was approximately normally distributed). For vitality of surviving individuals, we used the Kruskal-Walllis rank test, as it is appropriate for response variables with ordinal ranks. We also built generalized linear mixed models (GLMMs) including parent plant as a random effect. However, since parent plant was not significant, we report only the results of the models without this variable.

To further examine differences in responses between origins (i.e. geothermal vs. non-thermal lineages) and among heat treatments, we built multivariate models for each response testing the effects of heat treatment, origin and their interaction. We did not include responses following the cold and intermediate treatments in these models, due to potentially confounding effects of different temperature fluctuations, treatment durations and light conditions during those treatments. We did, however, test the influence of parent plant by comparing the sample-size corrected Akaike Information Criterion (AICc) of models with and without this variable included. As the AICc was not improved by including parent plant for any response (Table S2), this variable was not included in the final models. For the response variable survival, we built a generalized linear model (GLM) with a binomial distribution, treating survival as 1 and death as 0. The R² value was calculated as 1 - deviance/null.deviance, thus representing the proportion of the deviance explained by the model and indicating the percentage of the variance of the dependent variable explained by the independent variables. For the response variable biomass, visual inspection of the data indicated a hump-shaped relationship between treatment and biomass. Therefore, we fitted a generalized additive model (GAM) [73] using mgcv [74]. GAMs are a powerful approach for modelling flexible regression functions to test complex relationships, while still retaining relatively straightforward interpretation of model outputs [75]. The number of knots was set to three following the recommendations of the Generalized Cross-validation (GCV) method, which balances simplicity against explanatory power [74]. Visual inspection of our data also suggested that models with three knots provided a good fit. For the response variable vitality, we fitted an ordinal logistic regression model (OLR) using the “polr” function from the MASS package [76], as is suitable for ordinal data with different increments between ranks.

## Results

### Hypothesis (i)– Higher survival and performance of geothermal plants following heat treatment

**Fig. 2:**
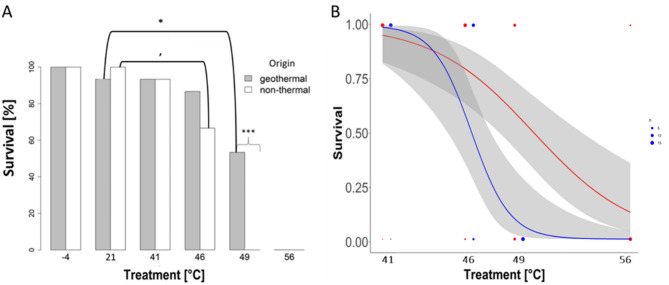
Survival rate of geothermal and non-thermal plants under different temperature conditions. **(a)** Percentage survival 30 days post treatment for geothermal (grey) and non-thermal (white) plants. Significant differences for between-origin tests are indicated by braces and significant differences for within-origin tests indicated by brackets. The significance level is indicated by asterisks (***p < 0.001, **p < 0.01, *p < 0.05, ’*p* < 0.1). **(b)** Predicted survival probabilities at different heat treatment temperatures based on a Generalised Linear Model (GLM) for plants of non-thermal (blue) and geothermal (red) origin. Confidence intervals are depicted in grey

***Survival***– All plants died following the 56 °C treatment (Fig. 2a), although two plants of geothermal origin showed initial signs of regrowth. Only geothermal plants survived the 49 °C treatment and there was a tendency for more geothermal plants to survive the 46 °C treatment (Fig. 2a), but there were no significant differences in survival between geothermal and non-thermal plants for the 46 °C and 41 °C treatments (Table 2a, Fig. S6). Almost all plants survived the 41 °C treatment, irrespective of origin (Table 2a, Fig. 2a).

**Table 2:** Pairwise comparisons between plants of geothermal and non-thermal origin for each treatment

a) Survival: Fisher’s exact test for geothermal vs. non-thermal plants							
Treatment [°C]		Odds ratio		*p*			
−4*		NA		NA			
21		0		1			
41		1		1			
46		2.30		0.65			
**49**		**inf**		**0.002**			
56*		NA		NA			

b) Biomass of surviving individuals: T-test for geothermal vs. non thermal plants							
Treatment[°C]	T		*p*				
−4	−1.41		0.17				
21	−0.84		0.41				
41	−0.27		0.78				
46	1.06		0.30				
49*	NA		NA				

c) Vitality of surviving individuals: Kruskal-Wallis rank sum test for geothermal vs. non-thermal plants							
Treatment [°C]	χ2		Df		*p*		
**−4**	**12.03**		**1**		**< 0.001**		
21	3.56		1		0.059		
41	2.50		1		0.11		
46	0.02		1		0.88		
49*	NA		NA		NA		

*Not possible to calculate as all (−4 °C), no (56 °C) or only geothermal (49 °C) plants survived

Results significant up to a level of p ≤ 0.05 are shown in bold

(a) Fisher’s exact test for survival

(b) T-test for biomass

(c) Kruskal-Wallis rank sum test for vitality

For both geothermal and non-thermal plants, survival rates were lower following the 49 °C and 56 °C treatments compared to the intermediate temperature (21 °C; Fig. 2a). However, only the 49 °C survival rates for geothermal plants could be tested against the 21 °C rates with the Fisher’s exact test, due to zero variance in survival for non-thermal plants at 49 °C and all plants at 56 °C (Table 3a, Fig. 2a). Survival rates were also lower for non-thermal plants at 46 °C than 21 °C (*P* = 0.10, Table 3a, Fig. 2a).

**Table 3:** Within-origin pairwise comparisons of responses to heat and cold treatments versus intermediate conditions

a) Fisher’s exact test for survival in each treatment vs. intermediate conditions				
Treatment [°C]		*P*		
−4 geothermal		1		
−4 non-thermal		1		
41 geothermal		1		
41 non-thermal		1		
46 geothermal		1		
46 non-thermal		0.10		
**49 geothermal**		**0.04**		
49 non-thermal*		NA		
56 geothermal*		NA		
56 non-thermal*		NA		

b) T-test for biomass for surviving individuals in each treatment vs. intermediate conditions				
Treatment[°C]	T		*P*	
−4 geothermal	−0.28		0.78	
−4 non-thermal	0.9		0.37	
41 geothermal	−1.72		0.1	
41 non-thermal	−1.85		0.08	
46 geothermal	1.61		0.12	
46 non-thermal	−0.18		0.86	
49 geothermal	−1.1		0.3	

c) Kruskal-Wallis rank sum test for vitality for surviving individuals in each treatment vs. intermediate conditions				
Treatment	X^2^		*P*	
−4 geothermal	0.41		0.52	
−4 non-thermal	0.41		0.52	
41 geothermal	0.12		0.73	
41 non-thermal	1.23		0.26	
46 geothermal	2.10		0.14	
**46 non-thermal**	**15.81**		**< 0.001**	
49 geothermal	2.86		0.091	

*Not possible to calculate as there were no surviving plants.

Results significant up to a level of *p* ≤ 0.05 are shown in bold

(a) Fishers exact test for survival

(b) T-test for test for biomass

(c) Kruskal-Wallis rank sum test for vitality

The multivariate model showed a significant effect of treatment (*P* = 0.002, R² = −0.006) but no effect of origin or their interaction (Table 4a). That origin was not a significant predictor in the multivariate model could be because its effect was strong at 49 °C but not at 41–46 °C, i.e., the significant difference found for 49° C in the pair-wise test is diluted in the multivariate models by the lack of differences at 41 and 46 ° C. Predicted survival probabilities based on this model clearly decreased with increasing temperature (Fig. 2b), with a tendency for survival rates to drop more sharply for non-thermal than geothermal plants at 46° C and above.

**Table 4:** Results of multivariate models assessing the effects of (heat) treatment, origin and the interaction between origin and treatment as predictors

(a) Generalized linear model for survival rate (*N* = 116, R² = −0.006, deviance explained = 0.5%)								
Predictor		Estimate		SE		t		*P*
Treatment		−0.86		0.27		−3.17		**0.002**
Origin		−16.07		14.24		−1.13		0.26
Treatment: Origin		0.377		0.30		1.26		0.21

(b) Generalized additive model for regrown biomass of surviving plants (*N* = 66, R² = −0.006, deviance explained = 4.5%)								
Predictor		Estimate		SE		t		*P*
Treatment		−0.5834		0.3870		−1.507		0.137
Origin		−18.394		20.2136		−0.91		0.367
Treatment: Origin		0.436		0.4622		0.94		0.350

(c) Ordinal logistic regression model for vitality of surviving plants (*N* = 66)								
Predictor	χ2		Pr(>|t|)					
Treatment	77.690		**0.002**					
Origin	0.996		0.38					
Treatment: Origin	35.952		0.06					

a) Generalized linear model for survival probability (binomial model)

(b) Generalized additive model for biomass of surviving plants (Gaussian distribution of square-root transformed biomass)

(c) Ordinal logistic regression for vitality

Significant results are shown in bold

The sign of the estimate depends on which group that was used as a reference

**Fig. 3:**
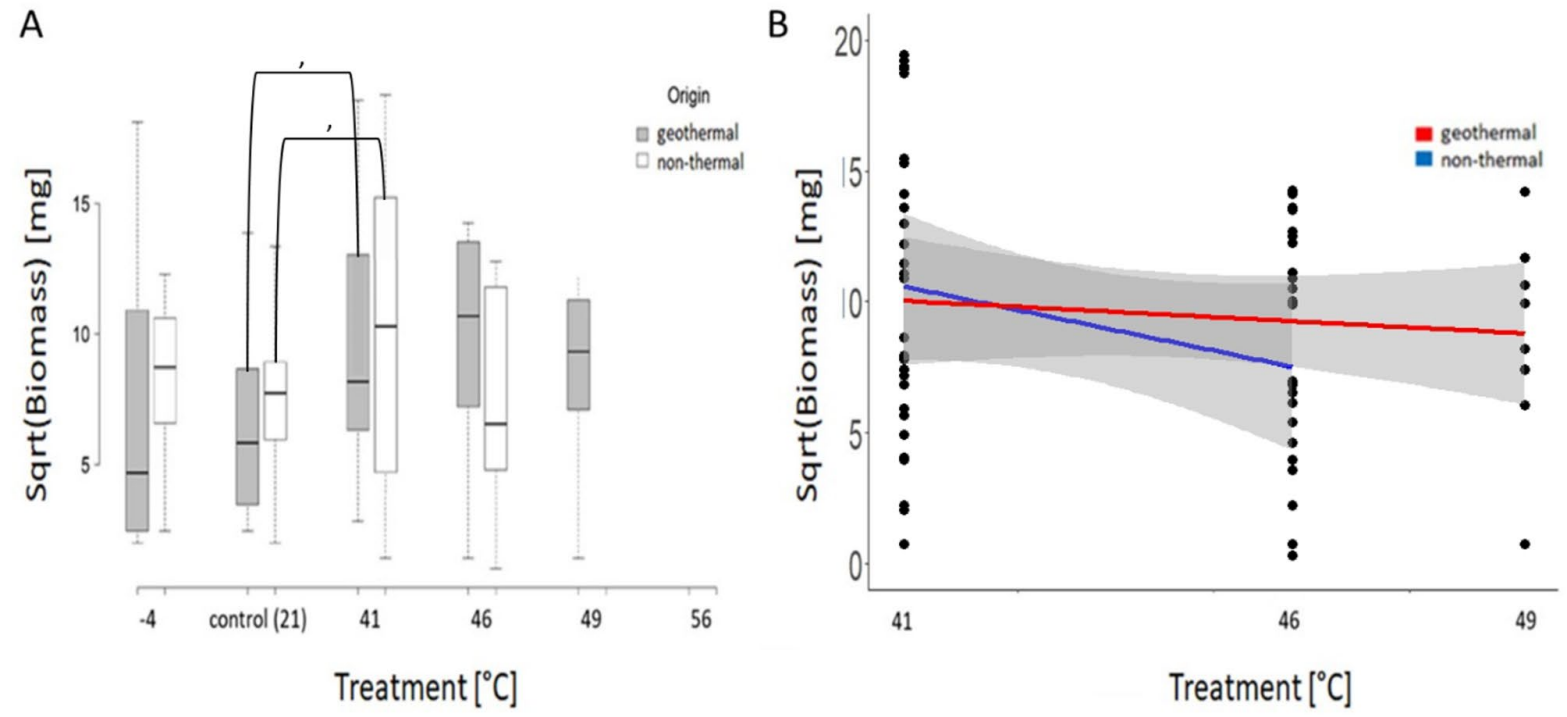
Biomass of geothermal and non-thermal plants under different temperature conditions. **(a)** Boxplot of the square root of the regrown biomass of surviving plants 30 days post treatment for plants of geothermal (grey) and non-thermal (white) origin. Box extent, 25th to 75th percentile; Band inside of the box, 50th percentile/median; Whiskers range, lowest to highest value within the 1.5 × inter-quartile range interval. The significance level is indicated by asterisks (***p < 0.001, **p < 0.01, *p < 0.05, ’*p* < 0.1). **(b)** Predicted square root of biomass based on a GAM model for plants from non-thermal (blue) and geothermal origin (red) for different heat treatment temperatures. Confidence intervals are depicted in grey

***Biomass***– No significant differences were found for biomass in the pairwise comparisons between geothermal and non-thermal plants following either the 46–41 °C treatments (Table 2b, Fig. 3a). Within-origin tests for heat treatment versus the intermediate temperature showed a tendency for both geothermal and non-thermal plants to produce more biomass at 41 °C than 21 °C (*P* < 0.1; Table 3b, Fig. 3a). The multivariate model showed no significant effect of treatment, origin or their interaction (Table 4b) and, accordingly, predicted regrowth from this model shows no clear relationship with temperature (Fig. 3b).

**Fig. 4:**
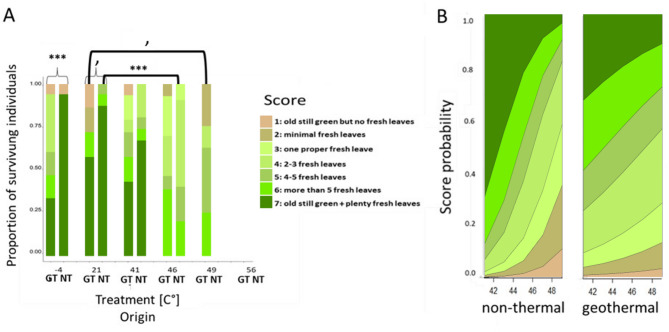
Vitality of geothermal and non-thermal plants under different temperature conditions. **(a)** Proportion of vitality scores from geothermal (left) and non-thermal (right) origin 30 days post treatment. Significantly different within treatment tests are indicated by braces and significantly different within-origin tests indicated by brackets. The significance level is indicated by asterisks (***p < 0.001, **p < 0.01, *p < 0.05, ’*p* < 0.1). **(b)** Predicted vitality probabilities based on an ORL model for plants from geothermal (left) and non-thermal (right) origin for different heat treatment temperatures. The score legend of **a)** also applies to **b)**

***Vitality***– There was no difference in vitality between geothermal and non-thermal plants following either the 46–41 °C treatments (Table 2c, Fig. 4a). Compared to plants kept at the intermediate temperature, non-thermal plants had significantly lower vitality following the 46 °C treatment (*P* < 0.001, χ2 = 15.81) and there was a tendency for geothermal plants to have lower vitality following the 49 °C treatment (*P* = 0.91, χ2 = 2.86; Table 3c, Fig. 4a). The multivariate model showed a significant effect of treatment (*P* = 0.002), no effect of origin but a tendency for an interaction (*P* = 0.06, Table 4c). Accordingly, predicted vitality scores decreased with increasing temperature, with a tendency to decline more strongly for non-thermal than geothermal plants (Fig. 4b).

### Hypothesis (ii)– Lower survival and performance of geothermal plants following cold treatment

All plants survived the cold treatment (Fig. 2a). There was no difference in biomass between geothermal and non-thermal plants following cold treatment (Table 2a, Fig. 3a) but vitality was significantly lower (*P* < 0.001, χ2 = 12.03) for geothermal than non-thermal plants (Table 2c, Fig. 4a). Within-origin tests showed no difference in survival, biomass or vitality between plants exposed to the cold treatment and plants that remained at intermediate conditions (Table 3).

### Hypothesis (iii)– Lower performance of geothermal plants under intermediate conditions

There was no significant difference in survival or biomass between geothermal and non-thermal plants that remained at the intermediate temperature (21 °C, Table 2a, b). However, geothermal plants had a tendency for lower vitality than non-thermal plants (*P* = 0.059, χ2 = 3.55, Table 2c, Fig. 4a).

## Discussion

There has been a considerable amount of research addressing plant cold and heat stress responses separately [13, 23, 24, 26, 29, 67, 77], but much less is known about the consequences of adapting to thermal extremes. In other words, less research has addressed the extent to which adaptation to one thermal extreme limits adaptation to the other thermal extreme (hot-cold trade-off) and/or reduces performance across a broader range of temperatures (specialist-generalist trade-off), and the prevalence in nature of thermal specialisation in response to the local environment [3, 22, 32–34]. We addressed this using two closely related lineages, one geothermal and one non-thermal, and tested for evidence of trade-offs involved in adapting to geothermal heat. Specifically, we tested whether the geothermal and non-thermal lineages of *Agrostis stolonifera* (Poaceae) in Iceland differ in their responses to heat and cold exposure, and tested the prediction that geothermal plants are more heat tolerant but perform worse under cooler conditions. In agreement with this prediction, our results showed that geothermal plants had higher survival and growth rates following exposure to the hottest temperatures, but performed worse at intermediate and cold temperatures. This suggests that the geothermal lineage is more heat tolerant and that this heat tolerance comes at a cost, reducing performance under cooler conditions. Overall, our results thus provide some support for the hot-cold tradeoff hypothesis, suggesting that the geothermal lineage could constitute a thermophilic specialist.

### Heat stress response

There were distinct differences in heat response between the geothermal and non-thermal lineages of *A. stolonifera*. The geothermal lineage was more heat tolerant than the non-thermal lineage: only geothermal plants survived the 49 °C treatment and there was no loss in performance at 46 °C compared to 21 °C, whereas non-thermal plants performed worse at 46 °C compared to 21 °C (Tables 2 and 3; Figs. 2, 3 and 4). The two measures of performance (growth ability), biomass and vitality, yielded similar results but the results for vitality were statistically significant. There was high variation in biomass, both within and among treatments (Fig. 3a), possibly as a result of some size variation at the start of the experiment, and the resulting models had low explanatory power (Table 4b). Vitality proved to be a less variable measure of performance (growth ability). Parent plant had no effect on survival or performance, suggesting that most of the observed variation occurs between the two lineages (Table S2).

Based on the survival rates measured here, the upper thermal limit for short-term heat exposure seems to lie between 49 and 56 °C for geothermal *A. stolonifera* and 46–49 °C for non-thermal *A. stolonifera*. These findings correspond with temperatures measured in the field: 49 °C was regularly measured at the geothermal but not the non-thermal area (Fig. S2). The poor performance of non-thermal plants at 46 °C and above is also in line with research showing that protein degradation and heat shock protein synthesis are induced in *A. stolonifera* at 40–45 °C [68], that temperatures above 40 °C are seriously harmful to most plants [52], with the upper thermal limit lying between 45 and 60 °C, including measures on both hardened and non-hardened plants [13, 29]. More extreme heat tolerances have been measured for Cactaceae, *Aloë* (Asparagaceae), Amaranthaceae and Zygophyllaceae [13]. Certain cacti and agaves, for example, have been shown to outlast 30 min at 60 °C, or even 65 °C, without harm [13, 78]. *Agrostis* contrasts with these arid-adapted tropical plants in that it inhabits temperate zones [55–57] and *A. stolonifera* occurs primarily in wet habitats [79]. Outside of geothermal areas, *Agrostis stolonifera* has not been recorded from very hot or dry habitats. Adaptations to heat in *A. stolonifera* are therefore unlikely to be ancestral, suggesting that the geothermal lineage has evolved adaptations to surviving the extreme temperatures of its specific habitat.

These adaptations may involve shifts in phenology, life history and habit or the plant’s inherent physiology. Geothermal *A. stolonifera* forms large, long-lived clonal mats, which flower more rarely and earlier during the season than the non-thermal lineage [45, 66, 80, 81]. These life-history differences have likely aided ecotype formation and maintenance of the geothermal lineage as distinct from the non-thermal one, because long-lived organisms may be more likely to accumulate mutations [82] and rare and earlier flowering is expected to reduce gene flow from surrounding populations [66]. Phenological and life history shifts, which promote reproductive isolation, have been found for other geothermal plants as well [49, 50], and may contribute to a general mechanism by which geothermal specialists originate and persist.

Another potential adaptation to the extreme temperatures of geothermal areas is the plant microbiome. A growing body of literature is demonstrating a central role of microbial symbionts in a range of plant stress responses [83–86]. One relevant example is the geothermal grass *Dichanthelium lanuginosum* found close to hot springs in Yellowstone National Park (USA), where rhizosphere temperatures reach 40–57 °C [87]. This species has been shown experimentally to survive over 10 days at 65 °C– but only in the presence of beneficial symbionts in the form of an endophytic fungus and a virus [40]. The proposed mechanism whereby the symbionts confer heat tolerance is that the virus limits the cell stress response and the fungus deals with harmful reactive oxygen species generated during the stress response. Microbial triggering of the plant’s own responses and/or sharing of metabolites, such as heat shock proteins, have also been suggested [86, 88]. We do not know whether such symbiotic relationships are important in our study system but it is likely given the increasing evidence for their importance for plant tolerance of a range of stresses, including elevated temperature [85, 86, 89]. Importantly, since the core microbiome is generally vertically transferred, it is possible that at least part of it was included in our experiment; plants were grown from field-collected seed without sterilization prior to cultivation. Further research is needed to elucidate the precise mechanism behind the increased heat tolerance of geothermal *A. stolonifera* demonstrated here and the potential role of any microbial symbionts.

### Cold stress response

Most previous research on geothermal soils and the plants that inhabit them has focused on heat tolerance and effects of adapting to warmer soils during the growing season [40, 44, 46–50]. Much less is known about how geothermal heat alters soil temperatures during winter and the extent to which geothermal organisms are able to tolerate low temperature stress. We found that all geothermal plants survived the cold treatment, demonstrating that geothermal *A. stolonifera* is able to survive short term cold and frost exposure. This is to be expected given the rather mild frost treatment, the generally high frost tolerance of the Poeae tribe, where *Agrostis* is found [67, 90, 91] and the general resilience of *A. stolonifera* to adverse conditions, including ice encasement [66]. However, we also found that geothermal plants had lower vitality than non-thermal plants following cold treatment. Thus, our results show that although geothermal plants are cold tolerant, they are negatively affected by short term cold and mild frost exposure, which non-thermal plants are not. These findings are in line with expectations based on the temperatures monitored in situ (Table 1), which revealed that geothermal *A. stolonifera* is occasionally exposed to sub-zero temperatures during winter but not summer, and that geothermal plants do not experience typical subarctic winters with seasonal cold and frost, but rather short cold/frost spells. Thus, geothermal plants need to have a permanent readiness for freezing in winter, while simultaneously contending with hot soil temperatures. This requirement for simultaneous readiness for high and low temperature stress might explain the reduced performance of geothermal plants following cold exposure.

### Performance at intermediate temperatures and trade-offs in adapting to thermal extremes

We found that geothermal plants had lower vitality than non-thermal plants when grown under intermediate temperatures within the optimal range for most plants. Geothermal plants also had higher performance following heat exposure and lower performance following cold exposure compared to non-thermal plants. Together, these findings are consistent with there being a trade-off between heat tolerance and performance at less or other extreme temperatures. Since heat is the main factor that distinguishes geothermal and non-thermal plant communities in the field [50, 92, 93] it is unlikely that factors other than temperature have caused the differences between the geothermal and non-thermal lineages found here.

Only a very limited number of plant species are able to grow in the hottest geothermal soils, suggesting that a high degree of thermal specialisation and specific adaptations are needed to survive there. It is therefore likely that the observed trade-off is a consequence of such heat adaptation being costly, reducing performance under cooler conditions. In lieu of full TPCs for the geothermal and non-thermal lineages, our findings suggest that increased heat tolerance of geothermal plants is associated with a TPC that is shifted toward warmer temperatures overall. This implies that the two lineages may be differently thermally specialised, in keeping with theoretical expectations under the hot-cold tradeoff hypothesis [3, 22, 33]. However, quantification of detailed TPCs for the two lineages would ultimately be needed to fully test both the hot-cold tradeoff and specialist-generalist hypotheses.

We are aware of only one other study that has compared the performance of geothermal organisms under both hot and cooler conditions. That study found that the permanent requirement for heat tolerance in the common pond snail, *Radix balthica*, from geothermally heated waters made them *less* tolerant of extremely hot temperatures compared to snails from colder habitats [37]. This suggests that the trade-off for the snails was that adaptation to constantly elevated temperatures reduced tolerance of heat extremes, whereas in our grasses the cost of tolerance of heat extremes was reduced performance under cooler conditions. In fact, the finding for the snails is as expected according to another hypothesised trade-off between tolerance to acute and chronic exposure to thermal stress [94]. In other words, the thermal limits tolerated by an organism are expected decrease with increasing duration of exposure. Although geothermal *A. stolonifera* must be adapted to chronic heating, we saw no evidence of this trade-off here. However, different experimental conditions, e.g. increasing or varying the duration of thermal exposure, may have revealed this.

### Limitations

The cold, intermediate and heat treatments differed not only in their temperature conditions but in some other aspects as well, e.g., different durations, temperature fluctuations and light conditions. Therefore, it is possible that plant responses to the different treatments are not solely due to the different thermal conditions they were exposed to. However, temperature is the main difference experienced by the two studied lineages in the field and, thus, we expect that any adaptive differences between them are mainly related to temperature. Nevertheless, our analyses and the discussion above are focussed on comparing plant responses to the different heat treatments (that were the same aside from levels of heat exposure) and comparing responses of the geothermal and non-thermal lineage to the same treatment (i.e. cold or intermediate treatment; Tables 2 and 3). Our conclusions are therefore not based on comparison of results generated under different experimental conditions.

Most plants experience their hottest temperature exposure during the day and in the growing season, i.e. high temperature coincides with high light intensity. Since our heat treatments were performed in the dark, the light induced heat response, i.e. the light induced zeaxanthin production from violaxanthin via the electron transport chain that prepares for improved thermotolerance [95] might not be triggered. The plant responses measured here could therefore have been different if the heat treatment had been conducted under high light intensity. Importantly, however, geothermal organisms are not only exposed to heat during the day, but at night too and, depending where in the world they occur, in winter as well. In the natural habitat of the plants studied here, temperature fluctuations are not primarily coupled to sunlight intensity, but rather to fluctuating geothermal activity. As our temperature measurements show, high temperatures can occur at any time of day or year, including during the long subarctic winter with little to no light (Fig. S2). We therefore do not expect light to be a necessary part of in the heat response of these plants. It is possible of course that the combination of heat and darkness reduced performance of the non-thermal plants. However, there is no evidence for this, as their performance dropped only at 46 °C, consistent with what is known about optimal growth temperatures in plants in general and grasses specifically [52, 67]. Future experiments under both light and dark conditions, including more intermediate and cold temperatures, as well as differing durations of temperature exposure, will shed more light on the details of the adaptations of these high latitude grasses to their geothermal habitat. In the meantime, the best explanation for the results found here is that the two lineages differ in their thermal adaptation, with each being adapted to its natural thermal habitat.

## Conclusions

Our results suggest that Icelandic geothermal *A. stolonifera* might constitute a specialised, thermophilic lineage. While increased heat resistance was not associated with reduced ability to survive mild frost, it was associated with reduced performance (growth) under both intermediate and cold conditions. This provides some support for the hot-cold specialist tradeoff, such that adapting to geothermal heat may have caused geothermal plants to have a TPC that is shifted toward hotter temperatures [3, 22]. Interestingly, we did not find evidence that exposure to chronic heat has reduced tolerance of short term heat exposure, as found for other geothermal organisms [cf. 37, 94]. Overall, these findings are consistent with the measured temperatures in geothermal areas, which regularly reached over 50 °C, as well as sub-zero temperatures in winter. In addition, and contra expectations based on theory [8–11, 34], it is interesting that we obtain results consistent with thermal specialisation in the more thermally variable of the two studied habitats– the geothermal study sites experience greater spatial and temporal thermal fluctuation both above and below ground.

Thermal specialisation likely constitutes a competitive disadvantage for the geothermal lineage in non-thermal areas. Conversely, the lower heat resistance of non-thermal *A. stolonifera* likely prevents it from colonizing heated soils. Differences in heat tolerance and the associated trade-offs might thus explain the persistence of two separate lineages of *A. stolonifera* in Iceland. The presence of two closely related intraspecific lineages in close geographic proximity opens the door to further research on the mechanisms underlying the increased heat tolerance, the potential role of a habitat-specific plant microbiome and the benefit and cost of thermal specialisation under different climatic conditions. Finally, our results highlight that adapting to increasingly extreme heat caused by climate change could come at a cost, limiting plant growth under cooler conditions.

## Supplementary Information

Supplementary Material 1

## Data Availability

The data supporting the findings of this study and code used in the analyses are archived in the Zenodo data repository under the filename: “Nuppenau, J.-N. (2024). Dataset and R-script for Article: Increased heat tolerance of geothermal plants at the cost of reduced performance under cooler conditions Zenodo. https://doi.org/10.5281/zenodo.11400108.
