# Supplementary material for: Increased heat tolerance of geothermal plants at the cost of reduced performance under cooler conditions
Source: BMC Ecol Evol. 2025 Aug 14;25:81. doi: 10.1186/s12862-025-02422-7 (PMC12351892; doi:10.1186/s12862-025-02422-7)
Supplement: Supplementary file 1 — Supplementary Material 1 [file 12862_2025_2422_MOESM1_ESM.docx]

Increased heat tolerance of geothermal plants at the cost of reduced performance under cooler conditions

Jan-Niklas Nuppenau, Johan Ehrlén and Aelys M. Humphreys

**Supplementary Materials**

**Table of contents**

**Supplementary Tables**

**Table S1**. Temperature conditions, duration, locality and sample size of the six temperature treatments.

**Table S2.** AICc of models with and without parent plant for all three response variables.

**Supplementary Figures**

**Figure S1.** Habitat of non-thermal *Agrostis stolonifera* in Iceland.

**Figure S2.** Temperature variation at the geothermal and non-thermal study sites.

**Figure S3**. Schematic of cultivation process from harvest of the stolons to the start of the treatments.

**Figure S4.** Temperature curves recorded during the treatments.

**Figure S5.** Humidity curves recorded during the treatments.

**Figure S6.** Experimental plants 30 days post treatment.

**Supplementary Tables**

**Table S1.** Temperature conditions, duration, light conditions, locality and sample size of the six temperature treatments.

| **Treatment** | **Temperature^1^** | **Duration** | **Light conditions** | **Locality** | **No. of parent plants (sample size)** |
| --- | --- | --- | --- | --- | --- |
| 1 | max 41 °C | 24h | 24h dark | oven | 15 H, 15 C |
| 2 | max 46 °C | 24h | 24h dark | oven | 15 H, 15 C |
| 3 | max 49 °C | 24h | 24h dark | oven | 14 H, 14 C |
| 4 | max 56 °C | 24h | 24h dark | oven | 14 H, 14 C |
| 5 | min -4 °C | 48h | 15h light, 9h dark | outside | 15 H, 15 C |
| 6 | 21 °C | 24h | 12h light, 12h dark | greenhouse | 15 H, 15 C |

**^1^**Details of the temperature development during each treatment are provided in Fig. S4. H = plant of geothermal origin, C = plant of non-thermal origin.

**Table S2.** Sample-size corrected Akaike information criterion (AICc) of models with and without parent plant for all three response variables.

| **Model/Response** | **Survival** | **Biomass** | **Vitality** |
| --- | --- | --- | --- |
| AICc (with parent plant) | 11.07 | 686.26 | 363.08 |
| AICc (without parent plant) | 8.70 | 684.19 | 360.73 |

**Supplementary Figures**


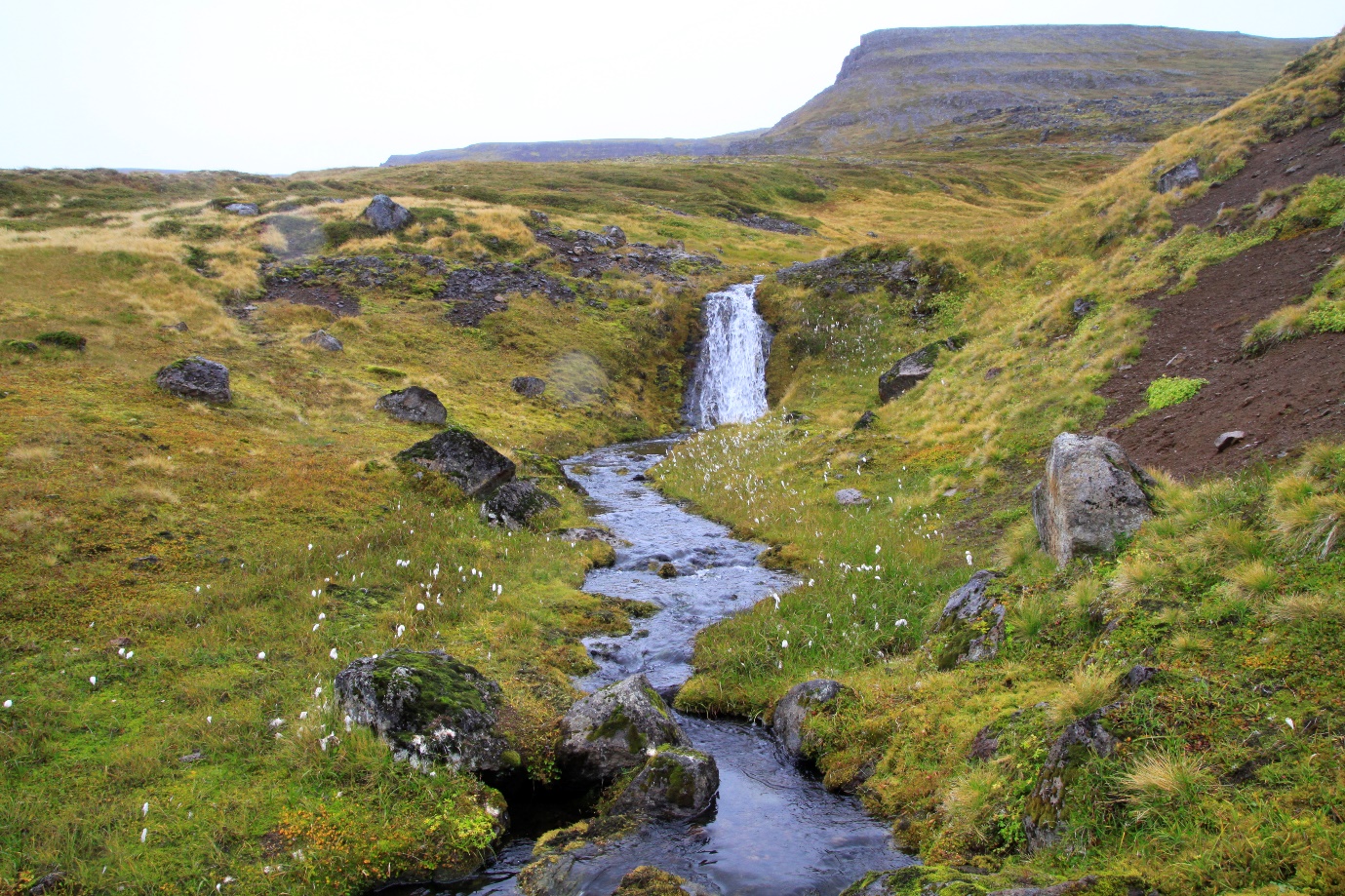


**Figure S1.** Habitat of *Agrostis stolonifera* at the non-thermal area in Iceland. *Agrostis stolonifera* mainly grows very close to the cold mountain creek seen here. Parts of the plants are constantly submerged in the cold creek water.

**
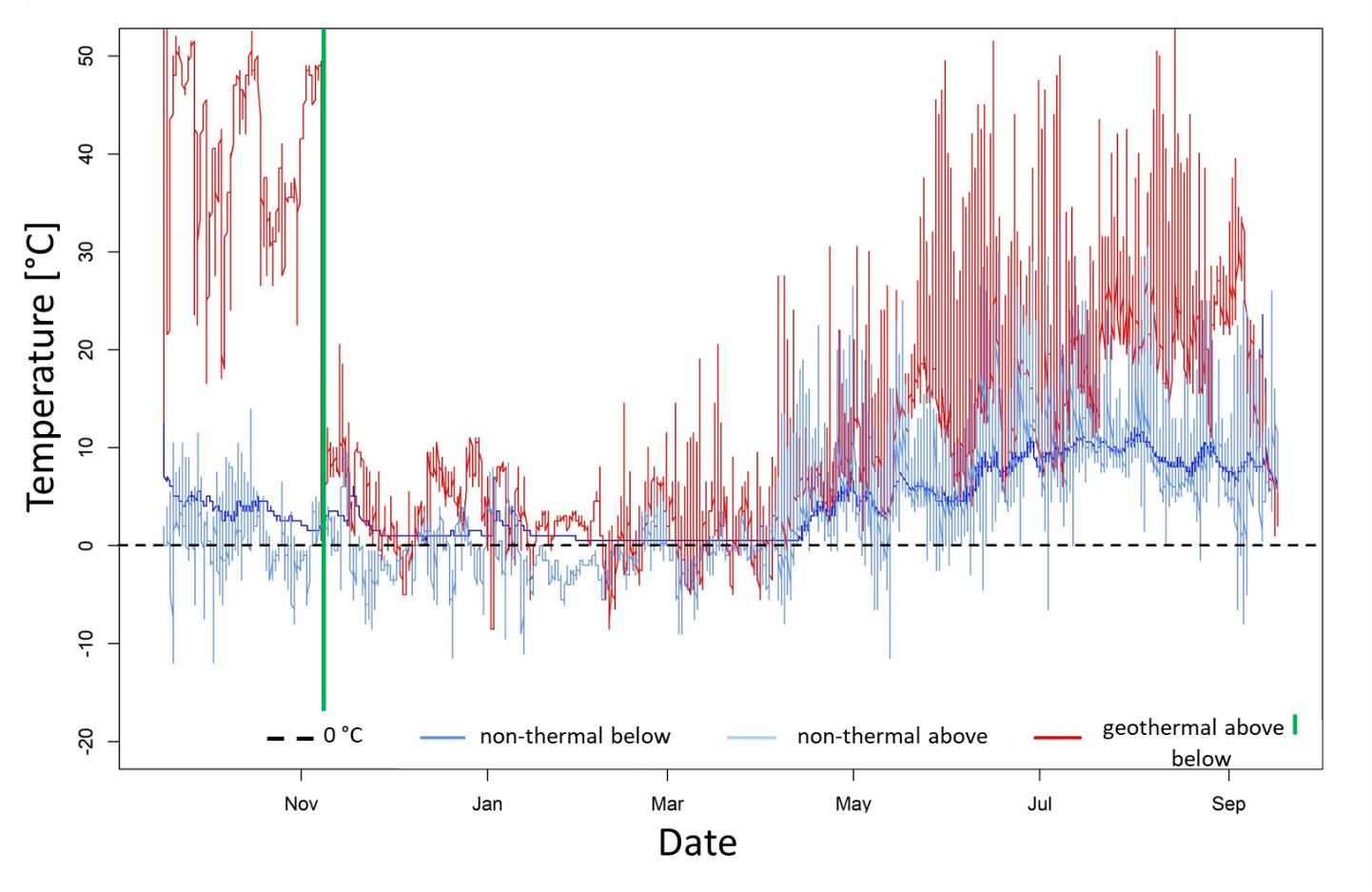
Figure S2.** Temperatures recorded every 2 hours at the geothermal and non-thermal area from 20 September 2018 to 14 September 2019. Stark diurnal temperature fluctuation results in the bar like appearance in places. Light blue: non-thermal site at the soil surface (aboveground), dark blue: non-thermal site belowground at 10 cm depth, red line: geothermal site, green vertical line: the timing of when the logger at the geothermal area was pulled out of the ground (5 Nov 2018), dashed, horizontal line: 0 °C.


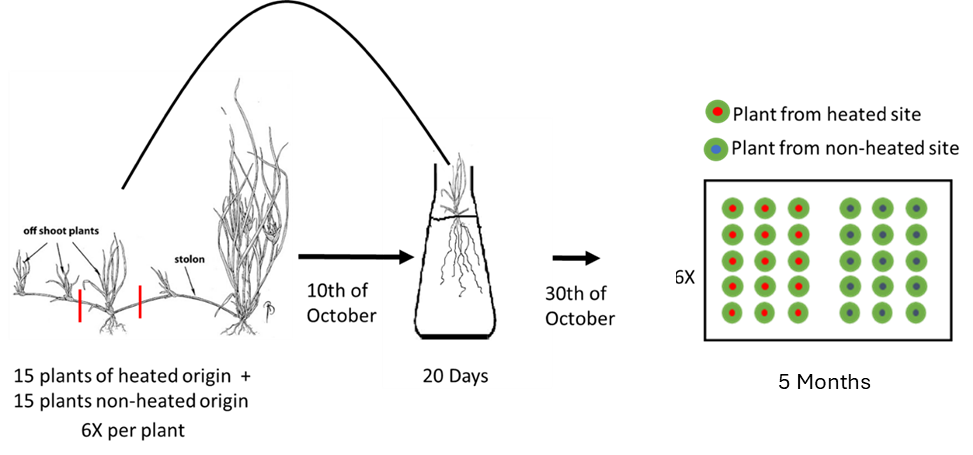


**Figure S3.** Schematic of cultivation process from harvest of the stolons to the start of the treatments. From 15 plants of thermal and 15 plants of geothermal origin respectively, six stolons were cut and transferred into water-filled containers, where they were kept for 20 days. Then, six stolons of a similar size from each of 15 geothermal and 15 non-thermal parental plants were transferred to trays containing 30 pots (diameter: 6.6 cm, height: 7 cm) filled with a mixture of sand and soil, where they were cultivated in the green house for 5 months until treatment. The six replicates are genetically identical and represent 15 different individuals (parent plants) from each origin.


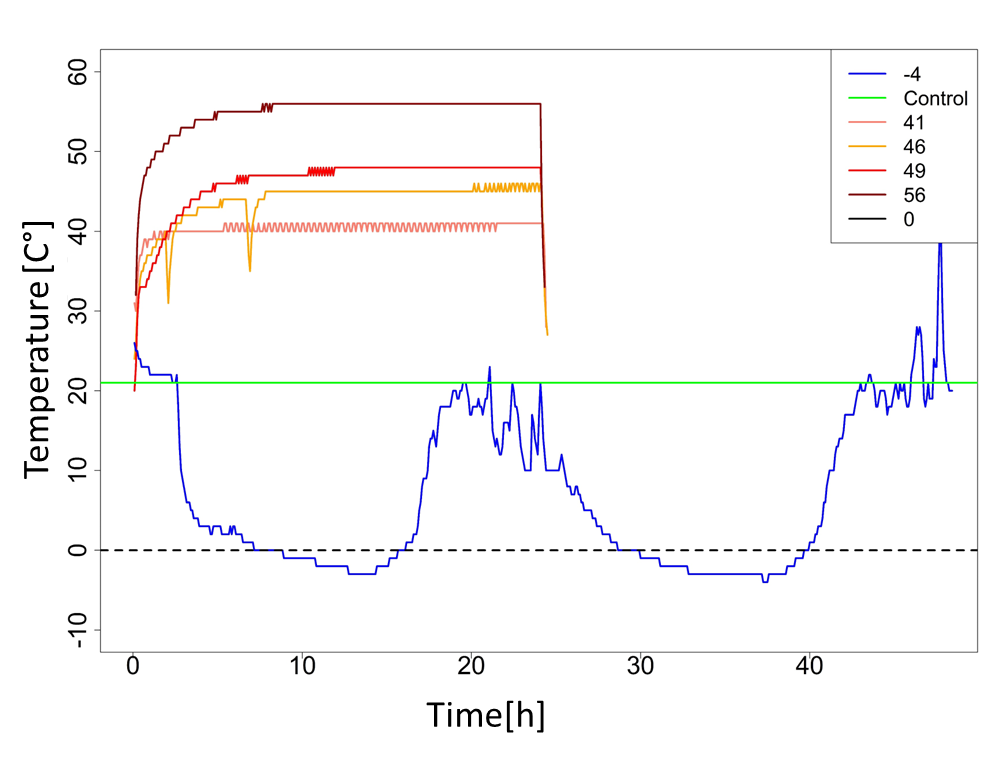
**Figure S4**. Temperature curves recorded during the treatments. Heat treatments: Solid rose, yellow, red and dark red lines represent the temperatures recorded in the oven over 24h for the 41, 46, 49 and 56 °C treatments, respectively. Cold treatment: The solid blue line represents the temperature recorded outdoors in Stockholm, Sweden on two consecutive nights in April 2021. Intermediate conditions: The solid green line denotes the temperature conditions in the greenhouse (control). The dashed black line indicates 0 °C.

**
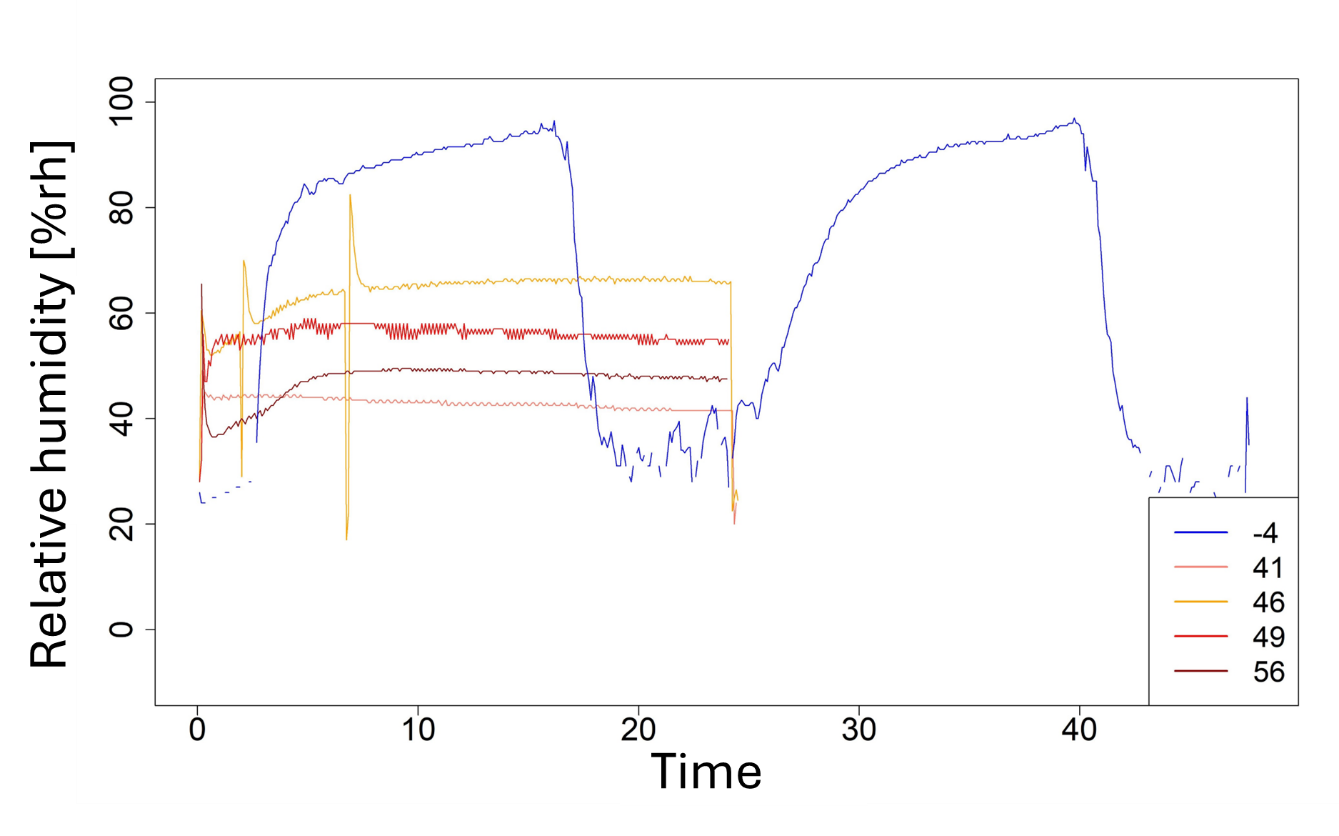
Figure S5**. Relative humidity curves recorded during the treatments. Heat treatments: Solid rose, yellow, red and dark red lines represent the relative humidity recorded in the oven over 24h for the 41, 46, 49 and 56 °C treatments, respectively. Cold treatment: The solid blue line represents the relative humidity recorded outdoors in Stockholm, Sweden on two consecutive nights in April 2021.


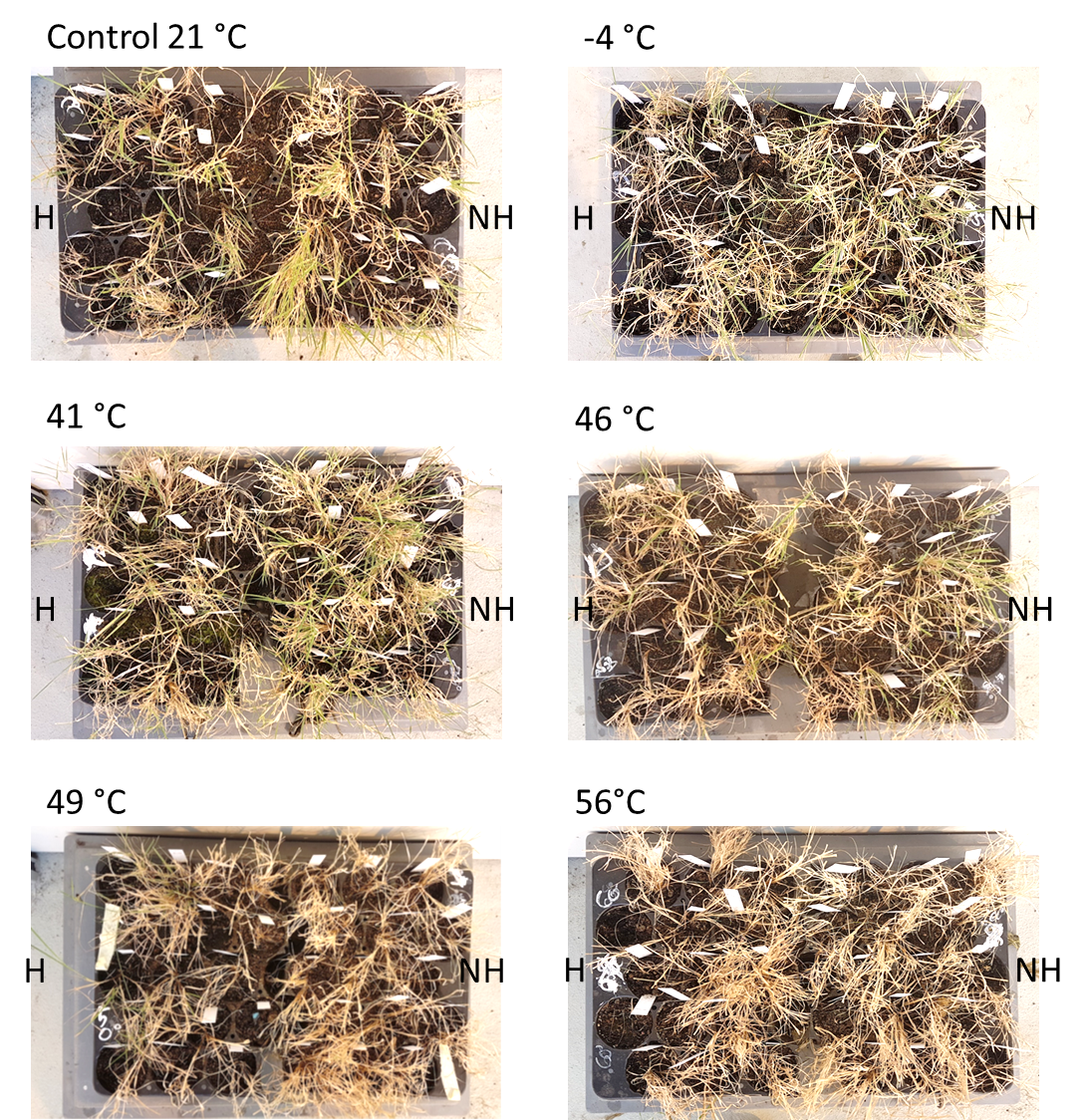


**Figure S6**. Plants following the 30-day post-treatment regrowth period in the greenhouse at 21 ºC. There were 15 plants of each origin (representing the geothermal and non-thermal lienages) in each treatment (H = geothermal origin/lineage; NH = non-thermal origin/lineage), except 49 and 56 °C for which there were 14 plants. The most notable differences are visible following the 49 °C-treatment, where green leaves can be seen in the geothermal plants but not non-thermal plants, and vice versa for 41 °C and below.
